# In vitro biodegradation of cyanotoxins in the rumen fluid of cattle

**DOI:** 10.1186/1746-6148-10-110

**Published:** 2014-05-08

**Authors:** Manjunath Manubolu, Samanthi RP Madawala, Paresh C Dutta, Kjell Malmlöf

**Affiliations:** 1 Department of Anatomy, Physiology and Biochemistry, Faculty of Veterinary Medicine and Animal Science, SLU, Box 7011 750 07 Uppsala, Sweden; 2 Department of Food Science, Swedish University of Agricultural Sciences, SLU 750 07 Uppsala, Sweden

**Keywords:** Biodegradation, Microcystins, Nodularin, Rumen microbial flora

## Abstract

**Background:**

In countries around the Baltic Sea grazing ruminants have access to and drink, surface water from lakes, rivers and in several coastal regions. The water quality of these naturally occurring reservoirs affects performance and health of livestock. In the Baltic Sea both microcystin (MC) and nodularin (NOD) occurs as cyclic peptides and have hepatotoxic effects. Although cattle obviously have died after consuming contaminated water very little information is available as to how susceptible ruminants are to the toxins produced by cyanobacteria. The critical question as to whether the rumen microflora might constitute a protective shield is unresolved. For this reason our aim is to investigate a possible degradation rate of these toxins in rumen.

**Results:**

The ability of rumen microorganisms to degrade certain important cyanotoxins (MC-LR, YR, RR and NOD) was studied in vitro by incubating with rumen fluid at three different concentrations (0.05, 0.5 and 5 μg/mL) for 3 h. The degradation efficiencies were determined by LC-MS (ESI) positive mode. Degradation was observed in the following order MC-RR 36%, NOD 35%, MC-RR 25% and MC-LR 8.9% at lower concentrations within 3 h. However, average degradation was observed at concentration of 0.5 μg/mL. No degradation was observed in higher concentrations for entire 3 h. The present results reveal that the degradation was both dose and time dependent.

**Conclusions:**

In conclusion the present results suggest that the rumen microbial flora may protect ruminants from being intoxicated by Cyanotoxins.

## Background

Toxins produced by cyanobacteria pose a worldwide threat to humans and animals due to their widespread occurrence in both fresh and sea waters [1,2]. Some freshwater strains of the *Microcystis, Oscillatoria, Anabaena,* and *Nostoc* species produce toxic compounds, termed microcystins (MCs) and nodularins (NODs) [3]. There are over 80 structural analogues of MCs, among which, microcystin-LR (MC-LR), microcystin-RR (MC-RR) and microcystin-YR (MC-YR) are the most common and extensively studied forms [4]. In addition to MCs, NOD is produced by a cyanobacterium *Nodularia* Sp. that is present in brackish waters like the Baltic Sea. It is a hepatotoxic pentapeptide and contains the amino acid residue Adda [5]. MCs are potent hepatotoxins [3] and tumor promoters [6]. They inhibit protein phosphatases 1 and 2A in hepatocytes [7,8], and may cause poisoning or death of many aquatic and terrestrial species including man [9-11]. Human exposure to MCs *via* drinking water may cause liver cancer [12], or acute deaths [11]. Recently, MCs were detected in the serum (average 0.228 ng MC-LR eq/mL) of chronically exposed fishermen [13], which indicate that low dose exposure may be common in certain segments of the population. World Health Organization (WHO) has set the provisional drinking water guideline value at 1.0 μg/L for MC-LR [14].

Animal exposure via drinking water contaminated with cyanobacteria was documented as early as 130 years ago [15]. Since then toxic water blooms have become more frequent and, people have become increasingly concerned about this potential hazard [16,17]. During the past decade, several Portuguese freshwater bodies, used for recreational and drinking purposes, have been found to have hepatotoxic blooms with production of diverse microcystins [18]. Worldwide the largest blooms of the toxin producing *Nodularia spumigena* mass events were recorded in the Baltic Sea [19].

These toxins are found inside the algal cells, but treatment of water with algaecides or natural death of cells result in release of the toxins in the surrounding water environment. Thus consumption of contaminated water is common cause to poisoning [20]. MCs will be transported across the ileal wall into the bloodstream using a bile-acid transporter that exists in the enterocytes lining the small intestine and in hepatocytes. The symptoms of intoxication such as hepatic damage in affected animals are similar irrespective of whether the toxin is given by intra peritoneal injection (i.p.) or oral administration [21]. However, animals are far less sensitive to MC-LR after oral, compared with peritoneal administration. This may be due to a certain breakdown executed by the gut microflora or mechanisms that inhibit absorption. It may therefore be suspected that the large microflora of the fore stomach (rumen) of ruminants may have a certain capacity to degrade algae toxins in the same way as has been shown for mycotoxins and hence constitute a first line of defense against toxic materials present in the diet [22,23]. Moreover, several mycotoxins and plant toxins have been shown previously to be detoxified by rumen microbes, ochratoxin A (OTA) and AFB1 [24].

However, little or nothing is known about microbial degradation of MCs and NOD under anaerobic conditions like those in the rumen of cattle. More knowledge in this field has both clinical and scientific relevance. For these reasons it would be interesting to study their stability and fate under the influence of microbial flora of rumen. To our knowledge, no one has previously addressed this issue. Therefore, the aim of the current work was to investigate the metabolic fate of cyanotoxins in the presence of rumen microflora of cattle to test the hypothesis that the microorganism of the rumen is capable of degrading toxins in a relatively wide dose range.

## Methods

### In vitro incubation

The whole rumen contents (250 mL) were collected from a fistulated Swedish red cow (Lövsta Research Station, Swedish University of Agricultural Sciences, Uppsala, Sweden) and transferred to the laboratory in pre-warmed thermo flasks. The animal was fed with hay and water was available *ad libitum* (Ethical permit number: C273/11 (valid from 2011-10-28 to 2014-10-28) issued by The Regional Ethical Committee (Uppsala djurförsöksetiska nämnd), Uppsala, and Sweden. The rumen contents were strained through a double layered cheese cloth and kept under CO_2_ flushing. All the tubes and measuring cylinders were gassed with oxygen-free CO_2_, screw capped until its use for incubation. After filtration, 1 mL of the whole rumen contents was taken in to glass tubes (triplicates) and each toxin (MCs LR, RR, and YR and NOD obtained from Sigma-Aldrich, St. Louis, MO, USA) was added at three different concentrations (0.05, 0.5 and 5 μg/mL). All the toxins were dissolved in distilled water. After flushing with CO_2,_ screw capped and incubated for 1, 2 and 3 h in a water bath with shaker at 39°C (Stalproducter, Uppasala, Sweden). In order to have the same volume (1.025 mL) in all tubes, 5–10 μL of water was added to the tubes. An additional 2 sets of triplicates were maintained, one containing only rumen fluid with water (without toxin) and second set as a 0 h control. After each incubation time, the reaction was stopped by adding 1 mL methanol. An exception was made for tubes at 0 h in which 1 mL methanol was added immediately after the toxin addition (without any incubation).

### Toxin extraction

After the incubation, the contents (rumen fluid, toxin and methanol mixture) were centrifuged for 10 min at 10,000×g (Eppendorf centrifuge). The supernatants (500 μL) were further filtered through ultra-free MC-Membrane filter unit (hydrophilic, PTFE, 0.20 μm, Millipore, Bedford, MA) for LC-MS analysis,

### Toxin analysis

#### Liquid chromatography electrospray ionization mass spectrometry

MC-LR, RR, YR and NOD were analyzed by LC-MS (HP 1100 Series, Agilent Technologies Inc., Palo Alto, CA) equipped with an autosampler, quaternary gradient pump, thermostatted column compartment kept at 40°C and single quadrapole mass analyzer (G 1946D) controlled by Chemstation Rev.B.04.01 software. Chromatographic conditions and parameters in MS were based on the methods of [25,26] optimized with modifications. A reversed phase Sunniest C18 column, 100 mm length × 2 mm i.d. and 5 μm particle size connected to a guard column SunShell RP, C18, 4×4 mm (ChromaNik Technologies Inc., Osaka, Japan), was used to analyze the toxins. The mobile phase used was composed of 0.1% formic acid in water (A) and acetonitrile (B). The gradient run was from 30% B to 65% B over 10 min, then to 30% B over 20 min. ESI -MS analyses was performed at the optimized settings; vaporizer temperature 350°C, drying gas temperature 350°C at a flow rate of 1.8 L/min, nebulizer pressure at 60 psi, corona current 8 μA, capillary voltage at 4000 V and fragmentor voltage at 70 V. Total ion current of mass spectra were recorded in the mass range *m/z* 100–1000.

For quantification, the mass spectrometer was operated in the positive ion mode to using selected ion monitoring (SIM) for MC-LR at m/z 498, YR at m/z 523, RR at m/z520 and for NOD at m/z825. The calibration curves of this method were constructed by injecting the standard solutions across 3 different concentrations (0.25, 2.5 and 25 ng/20 μL) for MC-LR, RR, YR and NOD. The rumen fluid was first tested to check matrix interference with toxin elution time. However no peak could be identified which had characteristic spectrum of toxins in rumen fluid. In all toxin degradation investigations, the percentage recovery of toxins was determined after the amount of toxins recovered at time 0. Subsequent toxin concentrations were adjusted to 100% recovery according to the determined percentage recovery at time 0.

#### Statistical analyses

Statistical significance between the groups was tested using the One-way ANOVA followed by Bonferroni’s multiple comparison as a post-hoc test. Calculations were executed in the GraphPad Prism 5.02 program (GraphPad Software, Inc., La Jolla, CA, USA). Mean values were considered different for *p* values less than 0.05, and data was presented as means together with standard error (SE).

## Results

Rumen microbial flora showed a capacity to degrade all four cyanotoxins. However, the degree of degradation was both dose and time dependent.

### Dose dependent degradation

**Figure 1:**
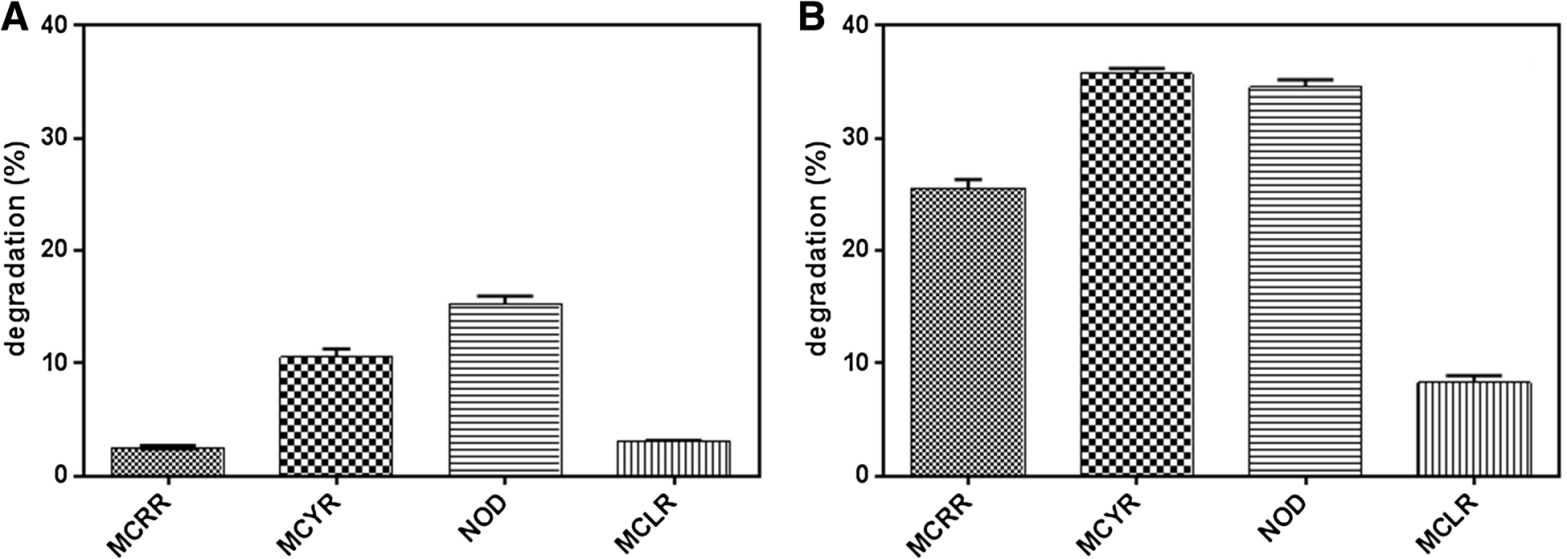
Dose dependent degradation of cyanotoxins (A) 0.5 μg/mL and (B) 0.05 μg/mL.

The highest degradation ratio was observed with MC-YR, showing a maximum removal of 36% (P < 0.05) at a concentration of 0.05 μg/mL (Figure 1B). This decreased down to 10% when toxin concentration was increased to 0.5 μg/mL (Figure 1A). No degradation was achieved at 5 μg/mL (data not presented). Thus, the degradation of MC-YR was shown to be dose dependent, with the highest percentage of degradation observed at low concentration (0.05 μg/mL); where as a substantial degradation was recorded for NOD with a maximum degradation of 35% (P < 0.05) at 0.05 μg/mL. In the case of medium concentration (0.5 μg/mL), maximum degradation was recorded as 18%. An average degradation was observed against MC-RR with rumen fluid as 25% at 0.05 μg/mL when compared with other three cyanotoxins. Very low and slow rates of degradation were observed as 3.2% at 0.5 μg/mL with MC-LR, whereas in the case of 5 μg/mL, no degradation was observed. The lowest degradation was observed against MC-LR when compared with MC-YR, MC-RR and NOD during at three different concentrations (Figure 1A and B). The maximum degradation was recorded as 8.9% at lower concentration which indicates the most resistant toxin among the four selected. No degradation was observed at 5 μg/mL.

### Time dependent degradation

The highest rate of degradation was observed as 36% during an incubation of 3 h with MC-YR.

**Figure 2:**
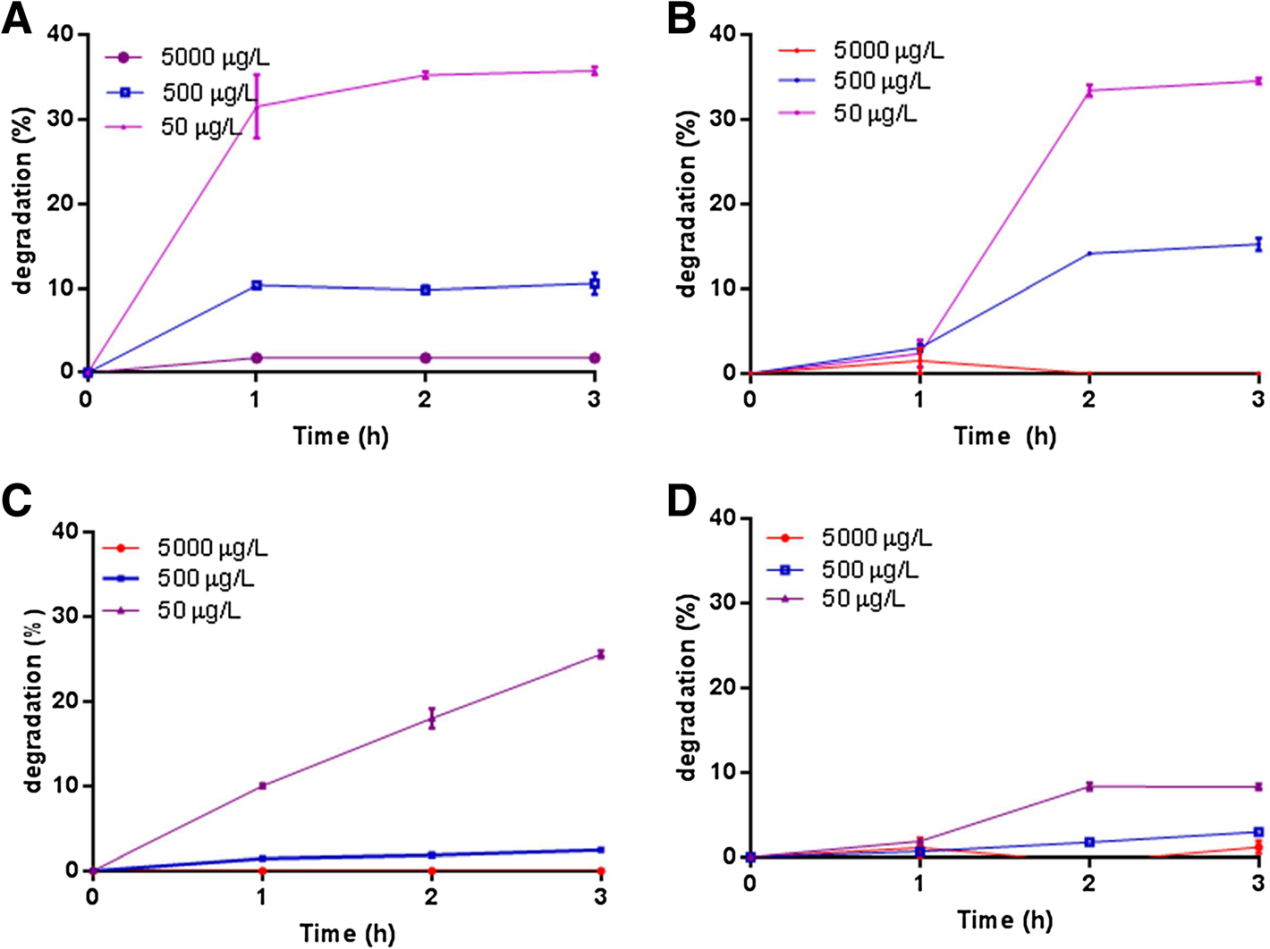
Time dependent degradation of cyanotoxins by rumen microbial flora within 3 h of incubation (A) MC-YR (B) NOD (C) MC-RR (D) MC-LR.

The degradation process was generally most rapid during the 1^st^ h and was successively decreased during 3 h incubation. In between the 1^st^ and 3^rd^ h a modest degradation of 6% (p < 0.05) of the added toxin was observed (Figure 2A). No further degradation was evident after 3^rd^ h. The maximum degradation of NOD was recorded as 35% (P < 0.05) during 3 h incubation. Already after one h of incubation, it reached up to 30% degradation and significantly (P < 0.05) it was further improved by 4% between 2^nd^ and 3^rd^ h (Figure 2B). No degradation was observed after 3rd h. This was true for all the three concentrations. The similar pattern of degradation was observed in the case of medium concentration (0.5 μg/mL) as 16% at 1^st^ h and 18% at 3^rd^ h. An average degradation was observed with MC-RR during 3^rd^ h (Figure 2C), whereas in the case of 5 μg/mL no degradation was observed within 3 h of incubation period. The degradation of MC-RR was increased along with the increased time up to 3 h, but further degradation was not evident after 3^rd^ h. Degradation was not initiated during first h, and very low percentage was observed at 2^nd^ h and no further degradation was observed after 2^nd^ h with MC-LR (Figure 2D).

## Discussion

In the present study, we investigated the degradation dynamics of the cyanobacterial toxins (MC-LR, RR, YR and NOD) under anaerobic incubation with rumen microbial flora. This is the first report in which cyanotoxins have been incubated with rumen fluid for degradation. In the present experiment, tested toxin range (0.05 - 5 μg/mL), to investigate the fate of cyanotoxins metabolism with the rumen fluid, is environmentally realistic.

World Health Organization (WHO) announced a tolerable daily intake (TDI) of MC-LR ingestion by human is 0.04 μg per kg body weight per day. No health effects or animal behavior was recorded on cattle when they injected 1.21 μg/kg body weight/d, which is 30 times higher than that recommend by the WHO as the maximum for oral ingestion of MC-LR by human [27]. The LD_50_ by intraperitoneal injection (i.p.) of MC-LR is about 50 μg*/*kg body weight. This toxin is 30–100 times less toxic via oral ingestion than via i.p. injection [28]. The oral route of administration has been prohibitively expensive for extended studies since the toxins are far less toxic orally than by the i.p. route. The *in vitro* methods provide an effective approximation of *in vivo* situations and have the advantage that reproducibility is good, as it is possible to control conditions better than in in vivo tests [29]. The *in vitro* incubation method is one of the best ways to investigate the fate of cyanotoxin metabolism when exposed to a broad range of concentrations.

In this study we have used one fistulated cow at our disposal as a representative of herd at SLU in Uppsala. As there is a little variation between rumen fluid microflora profiles of cows kept in the same environment. Further, the cow was maintained with the same feed and under the same conditions. Collection procedure was similar for the entire study. Similarly, in the previous studies also rumen fluid was collected from a single fistulated cow, which was used for measuring dissimilary reduction of nitrate and nitrite in the bovine rumen [30]. Kim et al. [31] also reported the dietary potential acid productions values, where rumen fluid was collected from a single fistulated cow. In the present investigation, concentrations of cyanotoxins (0.05, 0.5 and 5 μg/mL) incubated with rumen fluid are in correspondence to the sub chronic and acute doses of cattle announced by California Environmental Protection Agency [32]. The previous (*in vivo*) oral toxicity study [21,27,28] reveals the difference in sensitivity between oral and i.p. route of administration. In this association, the present results give a possible explanation as to why oral administrations are less toxic compared with parenteral. Our results suggest that the oral dose of toxin may be degraded when it passes through rumen before it reaches the intestine, blood circulation and liver. However, the percentage of degradation depends on dose and type of MC. The MC-LR is one of the most resistant ones when compared with MC-RR, MC-YR and NOD. The more sensitive one is MC-RR followed by NOD. The stability of the MCs in the *in vitro* incubation tests could be due to their chemical structures.

The degradation of cyanotoxins with rumen fluid was performed at three different concentrations for a period of 3 h at a time interval of 2 h. The time course degradation showed that the degradation depends on the type of toxin. No degradation was initiated during the initial 1^st^ h but after 1^st^ h, it reaches to the maximum around 2^nd^ h, further it was not improved in the case of MC-LR and NOD. But, in the cases of MC-YR and RR, the degradation was showed at initial 1^st^ h and further increased up to 3^rd^ h (data not shown) at low concentration. The time course was extended from 3^rd^ h to 8^th^ h for MC-RR and MC-YR at lower concentration to check for further degradation process, but further degradation was not evident after 3^rd^ h. This difference in the rate of degradation observed during the time course incubation may suggest that the degradation mechanisms are not the same.

Different bacterial strains, other than rumen microbial flora, have been shown to be able to degrade microcystins enzymatically. Bacterial degradation of microcystins has previously been reported for some *Sphingomonas strains*[5,33,34], a *Paucibacter strain*[35] and a *Pseudomonas aeruginosa strain*[36]. Recently, a bacterium named *Sphingosinicella* microcystinivorans was found to be able to degrade microcystins [37]. *Lactobacillus rhamnosus* strains GG and LC-705, *Bifidobacterium longum* 46, *Bifidobacterium lactis* 420 and *Bifidobacterium lactis* Bb12 were shown to be the most effective in toxin removal. One research report revealed that novel Clostridium clusters and their diverse consortiums dominate the bacterial communities during anaerobic degradation of microcystins [38] and another showed the enzymatic degradation of microcystin in the presence of probiotic bacteria [39]. All above biodegradation findings were investigated with in a period of 24–48 h.

The time required for the biotransformation of toxins which enter the body through digestive tract is also important; as some are degraded within short time and others need longer. In our study, *in vitro* incubation process was investigated with in a period of 1–3 h, which may be naturally a possible time for cyanotoxins metabolism in ruminants when exposed orally. MC-LR showed rapid absorption from the Gastro intestinal tract and metabolism of the parent compound within 1–2 h after oral administration in rat [40].

Cattle are usually poisoned when they drink from the windward side of these stagnant water bodies where the blue green algae have accumulated. In some cases, affected cattle die within a few hours of exposure; but in sub-acute cases, death occur later [41]. These *in vivo* observations were taken in to account, and we designed the *in vitro* experiment with a 3 h time bound period to investigate the metabolic fate of cyanotoxins under anaerobic condition with undiluted whole rumen fluid. We have monitored pH with in 3 hr for both low and high doses, and we did not find any notable variation. The degradation of MC’s could not be seen after 3 h incubation. In this case, after 3 h the microbial flora which is responsible for degradation might not be metabolically active or degradation process may need more time of incubation under anaerobic condition.

## Conclusions

The present studies of cyanotoxins degradation sheds new light on the ways by which cyanotoxins are degraded and can be useful in future exploitation of rumen microbial flora. The present results also reveal that the rumen microbial flora is capable of degrading the cyanotoxins. The reason as to why the rate of degradation slows down during the incubation in our *in vitro* system may be dependent on several factors. The metabolically active microbial population may be decreased due to loss of anaerobic conditions or accumulation of metabolic end products. In the case of higher and medium dose incubations, rumen bacteria may be inhibited by the toxin. From this point of view it may be possible to achieve complete degradation by further refinements of incubation conditions. In summary, our experiments have for the first time provided evidence for the biodegradation of cyanotoxins by the rumen microbial flora. The precise benefits and consequences of the present findings remain to be investigated *in vivo*. Future experiments may also be focused on increasing animal number, incubation conditions, including different pH conditions, to achieve complete degradation of cyanotoxins.

## Abbreviations

MCs: Microcystins; NODs: Nodularins; MC-LR: Microcystin-LR; MC-RR: Microcystin-RR; MC-YR: Microcystin- YR; WHO: World Health Organization; i.p.: Intra peritoneal injection; OTA: Ochratoxin A; Afaltoxins-B1: AFB1; MS: Mass spectrum; LC-MS: Liquid chromatography mass spectrometry; ESI: Electron spray ionization; SIM: Selected ion monitoring; SE: Standard error; TDI: Tolerable daily intake.

## Competing interests

The authors declare that they have no competing interests.

## Authors’ contributions

MMN assisted in conceiving the study, conducted analyses, and drafted the manuscript. SRPM assisted the analysis part. PCD assisted in the analysis plan and manuscript editing. KML, the principle investigator of the study, assisted in the analysis plan, results interpretation and manuscript editing. All authors read and approved the final manuscript.
